# Longitudinal trajectory of response to electroconvulsive therapy associated with transient immune response & white matter alteration post-stimulation

**DOI:** 10.1038/s41398-022-01960-8

**Published:** 2022-05-07

**Authors:** Blake Andreou, Benjamin Reid, Amanda E. Lyall, Suheyla Cetin-Karayumak, Antoni Kubicki, Randall Espinoza, Jennifer Kruse, Katherine L. Narr, Marek Kubicki

**Affiliations:** 1 Psychiatry Neuroimaging Laboratory, Department of Psychiatry, Brigham and Women’s Hospital, Harvard Medical School, Boston, MA USA; 2 Department of Psychiatry, Massachusetts General Hospital, Harvard Medical School, Boston, MA USA; 3 Ahmanson-Lovelace Brain Mapping Center, Department of Neurology, David Geffen School of Medicine, Los Angeles, CA USA; 4 Jane and Terry Semel Institute for Neuroscience and Human Behavior at UCLA, Department of Psychiatry and Biobehavioral Sciences, David Geffen School of Medicine, Los Angeles, CA USA; 5 Cousins Center for Psychoneuroimmunology, Los Angeles, CA USA; 6 Department of Psychiatry and Biobehavioral Sciences, University of California Los Angeles, Los Angeles, CA USA; 7 Department of Radiology, Brigham and Women’s Hospital, Harvard Medical School, Boston, MA USA

**Keywords:** Depression, Predictive markers, Neuroscience

## Abstract

Research suggests electroconvulsive therapy (ECT) induces an acute neuroinflammatory response and changes in white matter (WM) structural connectivity. However, whether these processes are related, either to each other or to eventual treatment outcomes, has yet to be determined. We examined the relationship between levels of peripheral pro-inflammatory cytokines and diffusion imaging-indexed changes in WM microstructure in individuals with treatment-resistant depression (TRD) who underwent ECT. Forty-two patients were assessed at baseline, after their second ECT (T2), and after completion of ECT (T3). A Montgomery Åsberg Depression Rating Scale improvement of >50% post-ECT defined ECT-responders (*n* = 19) from non-responders (*n* = 23). Thirty-four controls were also examined. Tissue-specific fractional anisotropy (FAt) was estimated using diffusion imaging data and the Free-Water method in 17 WM tracts. Inflammatory panels were evaluated from peripheral blood. Cytokines were examined to characterize the association between potential ECT-induced changes in an inflammatory state and WM microstructure. Longitudinal trajectories of both measures were also examined separately for ECT-responders and non-responders. Patients exhibited elevated Interleukin-8 (IL-8) levels at baseline compared to controls. In patients, correlations between IL-8 and FAt changes from baseline to T2 were significant in the positive direction in the right superior longitudinal fasciculus (R-SLF) and right cingulum (R-CB) (*p*_sig_ = 0.003). In these tracts, linear mixed-effects models revealed that trajectories of IL-8 and FAt were significantly positively correlated across all time points in responders, but not non-responders (R-CB-p = .001; R-SLF-p = 0.008). Our results suggest that response to ECT in TRD may be mediated by IL-8 and WM microstructure.

## Introduction

Major depressive disorder (MDD) is one of the most common and debilitating psychiatric illnesses in Western societies, with a lifetime prevalence estimated to be between 16 and 17% [1]. Associated with significant functional impairment, MDD is also the leading global cause of years lived with disability [2]. Despite the large number of therapeutic options available, between 15 and 33% of those with MDD suffer from treatment-resistant depression (TRD), such that two or more treatment attempts of adequate dose and duration have failed to result in remission of symptoms [3, 4]. For patients with TRD, electroconvulsive therapy (ECT)—the brief application of convulsive seizures using an electrical stimulus—is considered the gold standard of treatment, achieving remission rates of up to 50% [5]. Despite its efficacy, the precise mechanism by which ECT remediates depressive symptoms remains unclear [5].

Research has suggested that MDD is due, in part, to a dysfunction of the immune response, with MDD patients exhibiting higher levels of circulating pro-inflammatory cytokines in the peripheral blood compared to healthy individuals [3, 6]. The connection between MDD and an inflammatory state is further supported by several additional findings: (1) mood disorders are highly comorbid with somatic diseases that involve chronic inflammation, including diabetes mellitus and cardiovascular disease; (2) prolonged stress, a known risk factor for MDD, has been shown to induce a pro-inflammatory state; (3) use of pro-inflammatory cytokines as a therapy for somatic conditions, such as interferon-α for hepatitis C infection, is associated with an increased rate of MDD symptom development; (4) several anti-inflammatory therapies for autoimmune and inflammatory disorders have been shown to exhibit antidepressant effects; and (5) a number of available antidepressant medications possess anti-inflammatory properties [7–9].

Though its mechanism of action is not fully understood, ECT has been shown to induce a dynamic neuroinflammatory response. MDD patients undergoing ECT have generally been found to experience a transient spike in pro-inflammatory cytokines after a single session of ECT, which ultimately returns to baseline, or below baseline, following the course of treatment [3]. Van Buel et al. postulate that this acute increase in pro-inflammatory markers serves a neurotrophic role, stimulating changes in brain structure that precipitate clinical response [5]. More specifically, the authors propose that the initial increase in pro-inflammatory cytokines may stimulate the release of neurotrophins, such as BDNF, which may serve a neuroprotective role, such as the induction of hippocampal neurogenesis [5]. This hypothesis is consistent with previous studies reporting increased BDNF levels [10] and increases in hippocampal volume post-ECT [11, 12]. Furthermore, animal models have found volumetric increases in the hippocampus and a reduction in depressive behaviors in mice following an immune challenge, suggesting that immune-mediated neurotrophic changes may relate to therapeutic recovery in MDD [13]. Still, a number of antidepressant medications have been shown to increase BDNF, so it is possible that these changes alone do not account for the therapeutic efficacy of ECT [14].

In addition to altered immune activation, WM abnormalities have also been reported in patients with MDD [15, 16]. WM changes are frequently measured using fractional anisotropy (FA), the most well-established and widely used diffusion imaging measure of WM microstructure. A recent meta-analysis of 11 voxel-based DTI studies reported that MDD patients consistently exhibited reduced fractional anisotropy (FA) in tracts connecting the prefrontal cortex to other cortical and subcortical areas [17]. Though most studies did not differentiate between patients with MDD and TRD, there is a growing literature investigating WM alterations in patients whose depression is treatment-resistant [18]. When compared with healthy controls, those with TRD have been shown to exhibit a further reduction of FA in several brain regions, including the right anterior limb of the internal capsule, corpus callosum, bilateral external capsule, and fornix [19]. Among individuals with MDD, reductions in FA have also been shown to correlate with treatment resistance and symptom severity. When compared with first-episode MDD and healthy controls, de Diego et al. reported that those with TRD or chronic MDD exhibited significant reductions in FA across a number of WM tracts, including the cingulum bundle (CB), superior longitudinal fasciculus (SLF), inferior longitudinal fasciculus (ILF), and corpus callosum [18]. Moreover, symptom severity was inversely correlated with whole-brain FA in this population [18].

Few previous studies have investigated the effect of ECT on WM. Similar to the more widely studied effects of ECT on gray matter structure [12, 20], initial evidence suggests that ECT can also induce changes in WM microstructure. Specifically, Lyden et al. reported increases in FA pre to post-ECT across several dorsal fronto-limbic tracts, including the bilateral anterior CB, left SLF, and forceps minor [15]. Furthermore, the observed increases in FA following ECT were found to be associated with a response to treatment [15]. Another study investigating the effects of ECT in late-life MDD similarly found increases in FA across a variety of WM tracts sampled from ROIs in the temporal and frontal lobes [21]. However, since diffusion FA is sensitive to a wide range of microstructural changes, such as edema as well as myelin/axonal density/integrity [22], it remains unclear whether WM changes that occur after administration of ECT reflect true structural WM alterations [23], or if they are partially or wholly attributable to acute and transient inflammatory processes [5, 24].

Several advanced diffusion MR models have recently been developed to address the biological specificity of WM changes observed through dMRI. One of the more popular and potentially relevant for investigating ECT-induced WM changes in the free-water model. Free-water imaging distinguishes between an extracellular compartment—potentially reflecting edema, CSF contamination, and/or partial volume effects—and a cellular compartment proposed to be more reflective of underlying tissue microstructure. By separating out the partial volume effects of the extracellular compartment, this method enables the estimation of tissue-specific fractional anisotropy (FAt), which may better reflect longitudinal, neurotrophic changes in white matter microstructure that occur in response to ECT modulation.

In this study, we examine the relationship between levels of peripheral pro-inflammatory cytokines and potential longitudinal changes in WM microstructure following ECT in a population of TRD patients who did and did not respond clinically to ECT treatment. We hypothesized that ECT-induced inflammation modulates WM changes necessary for treatment response and that the longitudinal trajectories of pro-inflammatory cytokines and DMRI-indexed WM microstructure would therefore be aligned in TRD patients who ultimately responded clinically to ECT treatment.

## Methods

### Participants

Forty-two patients independently referred to receive ECT treatment were recruited from the University of California, Los Angeles (UCLA) Resnick Neuropsychiatric Hospital. Twenty-three (23) females and nineteen (19) males were enrolled in the study, with an average age at enrollment of 43.15 years (SD = 13.82 years). All patients had a DSM-IV-TR diagnosis of major depressive disorder, based on psychiatric evaluation and the Mini-International Neuropsychiatric Interview (M.I.N.I.) [25]. Exclusion criteria were the presence of comorbid psychiatric disorders or dementia, first-episode depression, the onset of illness after 50 years of age, depression related to a serious medical illness, and ECT or other neuromodulation therapies (vagal nerve stimulation, repetitive transcranial magnetic stimulation) within past 6 months. Patients were tapered off medications, including antidepressants, benzodiazepines, and anticonvulsants for a minimum of 48 to 72 h prior to enrollment.

All patients were diagnosed with treatment-resistant MDD (TRD), such that two or more antidepressant medication trials had previously failed to result in remission of the disorder [26]. Patients underwent a course of clinically prescribed ECT (5000Q MECTA Corp., Tualatin, Oregon) administered three times per week, using methohexital (1 mg/kg) and succinylcholine (1 mg/kg) for anesthesia and muscle relaxation. ECT followed the seizure threshold (ST) titration method wherein, after determining the ST (using a dose-titration method) at the first index session, ECT was administered at five-times ST for right-unilateral “d’Elia” placement using an ultrabrief pulse-width (0.3 ms), and 1.5-times ST for bilateral placement using a brief pulse-width (0.5 ms). All patients began with right-unilateral placement and, given a lack of efficacy with right-unilateral placement, transitioned to bilateral lead placement. This bilateral transition occurred in approximately 30% of our patients. On average, patients underwent 10.88 ECT sessions (SD = 3.91 sessions), with an average seizure threshold of 29.57 mC (SD = 25.94 mC) and seizure duration across all treatments of 67.77 s (SD = 19.46 s).

Patients were assessed at three time points over the course of ECT: within 24 h before their first session (baseline), following the second session occurring ~48 h after their first session (time point 2), and within 1 week of completing the course of treatment (time point 3). Depressive symptoms were evaluated using the Montgomery Åsberg Depression Rating Scale (MADRS) [27]. Subjects were defined as responders (*n* = 19) if their MADRS score improved by 50% or greater from baseline to second follow-up, and as non-responders (*n* = 23) if their MADRS score improved by less than 50% in this time frame.

Control subjects (*N* = 34, 39.44 years, 12.26 SD) had no history of any DSM-IV-TR disorder (confirmed by the M.I.N.I [25]) or prior use of psychotropic medications and were recruited to match TRD sample demographics, including age, sex, race/ethnicity, and level of education. Controls were assessed at baseline only. All participants provided written informed consent for study procedures approved by the UCLA Institutional Review Board.

### Blood-based measures of inflammation

Peripheral inflammation was assessed at each time point as previously described [24]. In brief, plasma concentrations of interleukin (IL)-6, IL-8, and tumor necrosis factor (TNF)-α were measured using a Bio-Plex 200 (Luminex Corporation, Austin, TX) instrument and a high-sensitivity multiplex immunoassay (Performance High Sensitivity Human Cytokine, R&D Systems, Minneapolis, MN). Data acquisition and analyses were performed with Bio-Plex software v4.1, and a 5-parameter logistic curve fit. Multiplex assays, which are shown to have high intra-assay reproducibility [28], were performed on samples diluted twofold as per the manufacturer’s protocol. C-reactive protein (CRP) was measured using Human CRP Quantikine ELISA (R&D Systems), also as per manufacturer protocols.

### Image acquisition

Two diffusion magnetic resonance imaging (dMRI) scans were acquired on a Siemens 3 T Allegra (Siemens Healthineers AG, Erlangen, Germany) for all subjects at each time point (baseline, time point 2, time point 3), and subsequently merged using an unbiased pairwise registration, which estimates optimal subject space and rotates gradients accordingly through affine registration. Merged DWI scans contained a total of 61 non-collinear diffusion directions with b = 1000 mm^2^/s, and 10 volumes without diffusion weighting (b = 0) (55 axial slices; TR/TE: 7300/95 ms; FOV: 96 × 96 over 24 cm; 2.5 mm isotropic voxels).

### Image processing

All dMRI scans were visually inspected for motion and signal loss, corrected for eddy current artifacts through affine registration to a b = 0 reference volume (FSL), and masked (using brain extraction tool (BET) plus manual editing) to remove non-brain areas. Free-water modeling and elimination was performed as described in Pasternak et al. 2009 [29]. This method separates out the partial volume effects of extracellular water and CSF on diffusion metrics, allowing for the estimation of tissue-specific fractional anisotropy (FAt) that is proposed to be more reflective of underlying tissue microstructure. First, fractional anisotropy (FA) and Free-Water (FW) maps were generated using preprocessed dMRI data. The FW fraction was subsequently removed from each voxel of the FA volume to generate free-water-corrected, tissue-specific FAt maps. Resulting FAt brain maps were registered to standard space (MNI space) following a two-step procedure to minimize mis-registration: first, a study-specific FA template was generated for each site using antsMultivariateTemplateConstruction2.sh; next, site-specific templates were linearly and non-linearly registered to the Illinois Institute of Technology (IIT) Human Brain Atlas FA map in MNI space using ANTs registration [30]. The affine and non-linear transformations were subsequently applied to register each individual subject (in template space) to the FA map in MNI space. The generated two-step transformations were then applied to FAt maps to warp them to MNI space.

### Statistical analysis

**Fig. 1:**
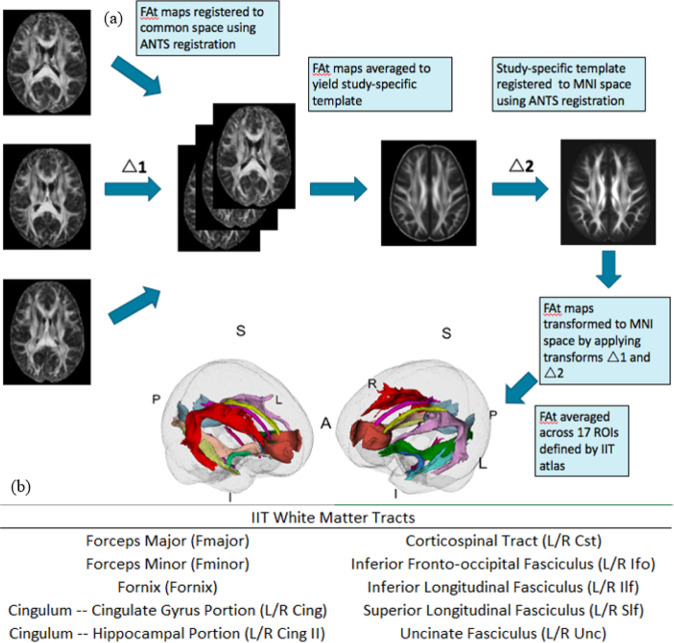
DMRI Processing Pipeline. **a** Registration of longitudinal FAt maps to IIT atlas and extraction of FAt; **b** List of utilized WM tracts as defined by IIT atlas (note L/R stand for left and right-hemispheric portions, respectively).

**Fig. 2:**
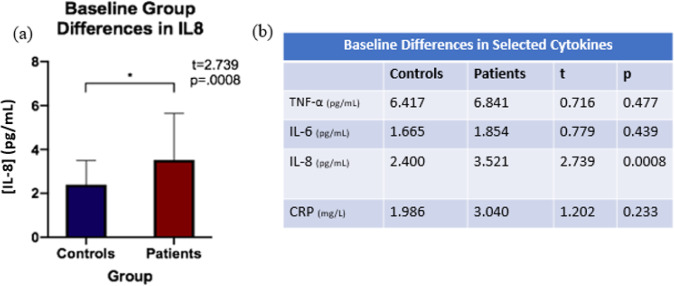
Baseline Cytokine Group Comparison. **a**Patient/control group baseline differences in IL-8 (error bars represent s.d.); **b** Patient/control Group differences at baseline in selected cytokines.

Independent sample *t*-tests were run to evaluate diagnostic group differences (individuals with TRD, controls) in cytokine levels at baseline (Fig. 1). Only cytokines showing significant differences between diagnostic groups at baseline were used for the investigation of relationships with change in tract FAt following ECT. Cytokines showing group differences at baseline (see Fig. 2) were selected for further analysis. To investigate biological correlates of alterations in cytokine activity in individuals with TRD compared to controls, Pearson correlations were run between the percent change of selected cytokines and percent change in FAt between baseline and time point 2 (i.e., ~48 h before treatment initiation and ~24 h after the second ECT session). It is important to note that changes between baseline to time point 2 were examined based on prior observations of an acute inflammatory response following the initiation of ECT that appears to normalize by completion of the ECT treatment series [3]. Bonferroni correction for multiple comparisons was performed for each of the above analyses to determine a threshold of significance (*p*_sig_). For tracts showing significant correlations between WM microstructure and inflammatory biomarkers, subsequent longitudinal analyses were performed.

A generalized linear mixed-effects model was used to investigate the relationship between longitudinal trajectories of cytokines of interest and FAt, separately in responders and non-responders, by following these steps: first, each selected cytokine was labeled as a response variable and was modified to be normally distributed using a log transform. Next, the modified cytokine level and FAt were standardized by z-mapping. Finally, we treated each subject as random effects in the mixed-effects model. Age and sex were included as fixed effects and Bonferroni correction for multiple comparisons was applied. Lastly, a cluster analysis was performed to potentially highlight trends in responders and non-responders that would not be apparent in group-level analyses.

## Results

### Subject characteristics

Patients and controls did not significantly differ in age (*p* = 0.20), sex (*p* = 0.96), or BMI (*p* = 0.16), but did significantly differ in education, with patients having a higher education level than controls (*p* = 0.03). Within the patient group, there were no significant differences between responders and non-responders in age (*p* = 0.16), sex (*p* = 0.14), or education (*p* = 0.20).

### Group differences at baseline

Patients showed increased IL-8 levels at baseline compared to controls, while the remaining cytokines (TNFα, IL6, CRP) were not significantly different between groups at baseline after Bonferroni multiple comparison correction (*p*_sig_ = 0.01) (Fig. 2).

### Longitudinal analysis

**Fig. 3:**
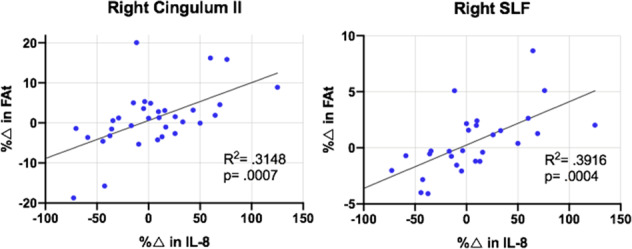
Correlations between FAt and IL-8. Correlations between percent change in FAt & IL-8 from time point 1 (pre-ECT) to time point 2 (immediately following ECT treatment) in R Cing II and R-SLF.

In patients, correlations between percent change in IL-8 and percent change in FAt from baseline to time point 2 were significant in right SLF and right cingulum II after correcting for multiple comparisons (*p*_sig_ = 0.003) (Fig. 3). In the left cingulum II and left uncinate fasciculus, there was evidence of trend level significance in these correlations after correcting for multiple comparisons (*p*_trend_ = 0.006).

**Fig. 4:**
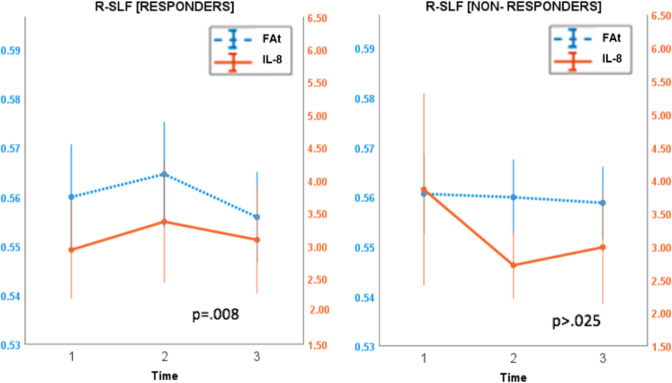
Cluster plots by response. Cluster plots revealed that responders were more likely to experience both a larger and a more positive change in FAt and IL-8 compared to non-responders.

Although IL-8 and FAt in right cingulum II and right SLF did not change significantly from baseline to time point 2 in either response group, responders qualitatively exhibited a larger and more positive average change in both measures compared to non-responders (Fig. 4). From baseline to time point 2, IL-8 increased by an average of 20.39% in responders and decreased by an average of 15.44% in non-responders. In the right cingulum II, responders experienced an average increase of 2.97% in FAt, while non-responders experienced an average decrease of 0.43% from baseline to time point 2. In the right SLF, FAt increased over this time by an average of 0.72% in responders and decreased by an average of 0.046% in non-responders.

**Fig. 5:**
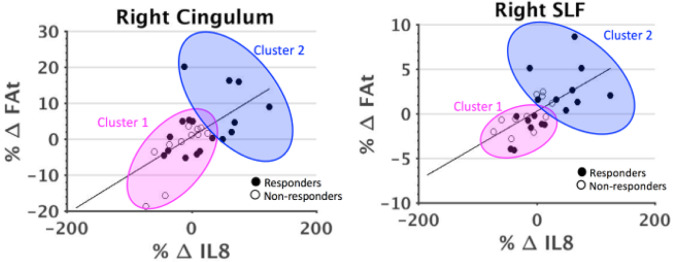
Trajectories of IL-8 and FAt in selected tracts. Using a linear mixed-effects model, the longitudinal trajectories of IL-8 and FAt were shown to be significantly correlated in responders in the Right SLF (above) after multiple comparison correction (*p*_sig_ = 0.025) (error bars represent s.d.). These trajectories were not correlated in non-responders. A similar trend (not shown) was observed in the right Cingulum II.

Using a linear mixed-effects model, the longitudinal trajectories of IL-8 and FAt were shown to be significantly correlated across all time points in responders in the right cingulum II (*p* = 0.001) and right SLF (*p* = 0.008) after multiple comparison correction (*p*_sig_ = 0.025). These trajectories were not significantly correlated in non-responders (Fig. 5).

## Discussion

This is the first study to assess the relationship between longitudinal, dynamic changes in WM integrity and levels of pro-inflammatory cytokines in the context of ECT. This study further builds on the relatively limited literature investigating effects of ECT by analyzing patients separately based on clinical response and contributes to the growing literature investigating WM microstructural changes in patients with TRD. In this study, we found diagnostic group differences in IL-8 at baseline, as well as a significant correlation between percent change in IL-8 and percent change in FAt in patients from baseline to time point 2 (i.e., 24 h after the second session) in the right cingulum II and right SLF. In these tracts, we additionally found that in patients who responded clinically to ECT, but not in non-responders, the longitudinal trajectories of IL-8 and FAt were significantly correlated across all three time points.

The pro-inflammatory cytokines most commonly reported to be elevated in MDD, including IL6 and TNF-alpha [3], were not statistically different between patients and controls in our sample. While there have only been a small number of studies focusing on IL-8 differences in MDD, a meta-analysis of immune activation in MDD reported that in all four studies that analyzed this cytokine, reduced levels of IL-8 were found in the blood of MDD patients compared with healthy controls [31]. However, within the MDD patient population, elevations in pro-inflammatory markers have been shown to positively correlate with treatment resistance, and a recent study analyzing cytokine profiles in patients with TRD found higher levels of IL-8 in patients compared to controls [8, 32]. In the context of existing literature, the baseline elevation in IL-8 levels observed in our sample of TRD patients may suggest that there is a different cytokine profile in patients with TRD than there is in the general MDD population.

While the association between ECT and changes in pro-inflammatory cytokines have been reported in several studies [7–9], the effects of ECT on levels of IL-8 are less well-established. IL-8 is a chemokine that is known to elicit widespread recruitment and activation of neutrophils in response to inflammatory stimuli [33]. In patients, the observed baseline elevation in IL-8 supports previous studies that have found an association between MDD and a peripheral inflammatory response [3]. Furthermore, the changes in IL-8 seen in our study are consistent with the hypothesis that ECT-induced neuroinflammation may serve a compensatory role, as IL-8 has previously been found to exhibit neurotrophic effects. Indeed, IL-8 has been shown to enhance neuronal survival of hippocampal cell cultures [34], and to promote neuronal growth following brain injury in TBI by stimulating the production of nerve growth factors [35]. It is therefore plausible that in responders, dynamic changes in IL-8 following ECT stimulate neuroprotective processes, such as glial proliferation, which promote clinical response to treatment. This potential glial proliferation may manifest as the transient increases in FAt that we observed in ECT-responders. In fact, rodent studies have established that glial cells comprise a substantial portion of WM voxel volume [36] and that alterations to both the quantity and organization of these cells can affect diffusion measurements [37]. In a recent publication, Sydnor et al. hypothesized that the transient FAt increases observed following ketamine infusions in TRD patients may be in part due to increased WM glial cell reactivity [38]. The hypothesized mechanisms by which ketamine infusions produce antidepressant effects often include immune modulation and subsequent alterations to glial cells, namely microglia and astrocytes [39]. Thus, our study provides some evidence that successful response to ECT may be immune-mediated in a similar fashion. This may suggest that successful response to multiple neuromodulatory techniques in TRD patients occurs via neuroinflammation-mediated glial activation, but more research will be needed to properly link the increase in pro-inflammatory cytokines and the increase in WM integrity.

It is not insignificant that the right cingulum II and the right SLF were the tracts most significantly associated with IL-8. The cingulum bundle has long been theorized to play a critical role in emotional regulation [40] and, while not directly tied to emotional processing, the right SLF has been reported to play a role in affective behaviors, such as empathizing and brooding [41, 42]. Previous research has demonstrated that those with TRD exhibit reduced FA in both the SLF and cingulum bundle [43], and that reduced FA in these tracts is associated with increased symptom severity and treatment resistance in those with MDD [18]. It is therefore plausible that immune-mediated processes serve to partially reverse structural deficits seen in the context of TRD. However, it is critical to acknowledge a second potential cause in the right-side bias we observed: all patients undergoing electroconvulsive therapy in the current study received treatment using either right-unilateral or bilateral lead placement. In other words, all subjects received some right-hemispheric stimulation, but only a subset of our population received stimulation on the left hemisphere. It is therefore also possible that our right-sided bias is a result of the lead placement parameters chosen as part of our population’s clinical treatment course. Further research should strive to further investigate the potential right-side bias of the current results, as well as the individual tracts implicated.

A strength of this study is the use of a highly dimensional, longitudinal dataset that permits analysis of biomarkers across the entire course of treatment. Additionally, the longitudinal nature of this study enables us to capture associations between the larger trajectories of these measures that would be obscured in cross-sectional analysis. It is important that future research into the effects of ECT reflects the longitudinal nature of the treatment paradigm, analyzing dynamic changes that occur over the course of treatment. An additional strength of our study is the use of free-water correction. Free-water imaging differs from standard diffusion tensor imaging as it utilizes a bi-tensor model to approximate a cellular “tissue” compartment and an extracellular “free-water” compartment. Identifying and eliminating the “free-water” creates a depiction of the WM microstructure independent of the extracellular compartment. This facilitates the detection of microstructural changes beyond alterations to WM, which in turn allows for a more precise and nuanced analysis of microstructural changes than would be possible with the standard fractional anisotropy measure.

There are several limitations to this study. First, due to the observational nature of this study, it is not possible to determine whether IL-8 changes precipitate alterations in WM, and therefore a causal relationship cannot be deduced. Second, given the sparse amount of research into the immune-activating potential of IL-8, further investigation is needed to determine its potential role in mediating white matter changes related to treatment response in ECT. Finally, the small sample size and high variability within the sample precluded the detection of statistically significant changes in FAt and IL-8 between discrete time points in either response group. However, our additional clustering analysis suggests that responders were more likely to experience a larger and more positive change in FAt and IL-8 compared to non-responders. Qualitatively, our results suggest that the magnitude of these changes differs between responders and non-responders, but we are unable to demonstrate these changes statistically. This may be in part due to the small sample size and high variability within the sample.

In summary, the current study examined the relationship between pro-inflammatory cytokines and WM changes throughout a course of ECT, in a population of patients diagnosed with TRD. An association between the longitudinal trajectories of IL-8 and FAt across two tracts was observed in those who responded to ECT treatment, elucidating a potential role of IL-8 in mediating WM changes that predict treatment response. Future endeavors should strive to further characterize the relationships between IL-8 and WM tracts throughout the ECT course, focusing on potential trajectory variations between responders and non-responders.
